# Editorial: Insights in drug metabolism and transport: 2021

**DOI:** 10.3389/fphar.2023.1198598

**Published:** 2023-05-09

**Authors:** Janny Piñeiro-Llanes, David E. Stec, Rodrigo Cristofoletti

**Affiliations:** ^1^ Center for Pharmacometrics and System Pharmacology at Lake Nona (Orlando), Department of Pharmaceutics, College of Pharmacy, University of Florida, Gainesville, FL, United States; ^2^ Department of Physiology and Biophysics, Cardiorenal and Metabolic Diseases Research Center, University of Mississippi Medical Center, Jackson, MS, United States

**Keywords:** transport, metabolism, organoids, PBPK, microphysiologic system

## Pharmacokinetics-related attrition rate in drug development

Improving drug translation is becoming a top priority as research and development costs keep increasing. It typically takes 10–20 years and a couple of billion dollars to bring a new treatment from initial discovery to final approval (Wouters et al., 2020; Schlander et al., 2021). In this process, as much as 90% of the novel drugs fail the first two phases of clinical approval, frequently called “the valley of death” (Takebe et al., 2018; Dowden and Munro, 2019). By 1991, adverse pharmacokinetic (PK) and bioavailability results, including toxicity and treatment failure, were the most significant cause of attrition, and accounted for ∼40% of all attrition in drug development. In turn, a retrospective analysis of the 148 failures between Phase II and submission in 2011 and 2012, revealed that less than 1% was due to PK reasons. These data illustrate the significant improvement in the translatability of PK properties at early stages of drug development. In fact, interspecies allometric scaling of PK parameters is commonplace in drug development. For example, for all species, values of systemic clearance (Cl), distribution clearance (ClD), central volume of distribution (Vc), and volume of distribution at steady-state (Vss) were highly correlated (*r* ^2^ = 0.89–0.99) with body weight. Furthermore, physiologically-based pharmacokinetic (PBPK) modeling improved the translatability of PK parameters derived in preclinical models by accounting for physiological differences between species.

Theoretically, it would be easier to translate from *in vitro* to *in vivo* systems (e.g., intra-species but between systems) than between species (but intra-system). For example, at early stages of drug development, metabolic clearance is initially estimated using rat liver microsomes. *In vitro-in vivo* extrapolated liver metabolic clearance in rats is then compared with estimates from *in vivo* studies in rats. In case the translatability is successful, scientists would estimate human liver metabolic clearance from human liver microsomes. In fact, this approach has been advocated by the National Center for Advancing Translational Sciences during the “*Microphysiological Systems: Bridging Human and Animal Research—A Workshop*” (Austin, 2021).

Unfortunately, the lack of physiological relevance of conventional *in vitro* cell culture systems models used in preclinical studies has historically warranted the need for animal models. Nevertheless, developing *in vitro* reductionist models emulating organ-level structure and functionalities is an evolving field that has significantly matured over the past 4 decades (Virumbrales-Muñoz and Ayuso, 2022). This field might help bridge the gaps in drug clinical translation and provide a better understanding of between-subject variability. For instance, organoids and microphysiological systems (MPS) are two emerging models to recapitulate key organ elements. These models, together with cell reprogramming induced pluripotent stem cells (iPSCs), are promising approaches to improve the prediction of PK parameters at early stages of drug development. This approach is also in line with the policies to reduce, refine, and replace animal use in research and is expected to play a major role in the future of drug development.

## Microphysiological systems: tool with the potential to improve clinical translation on new drugs

Organoids, usually generated from primary tissue cells or stem cells, are miniaturized 3-dimensional structures of multiple cell layers that recapitulate the organ’s anatomical microstructure. Because organoids can be created from patient-specific multipotent SCs or iPSCs, they are a valuable platform for drug screening and clinical precision treatment (Matsui and Shinozawa, 2021; Cho et al., 2022). For instance, patient-specific intestinal organoids have allowed for correlating cystic fibrosis (CF) pathogenic variants with variations in the clinical severity of the disease (Van Mourik et al., 2019). In fact, organoids are a robust platform that could help screen for therapeutic strategies to treat rare pathogenic variants as well as to prospect between-subject variability.

**FIGURE 1:**
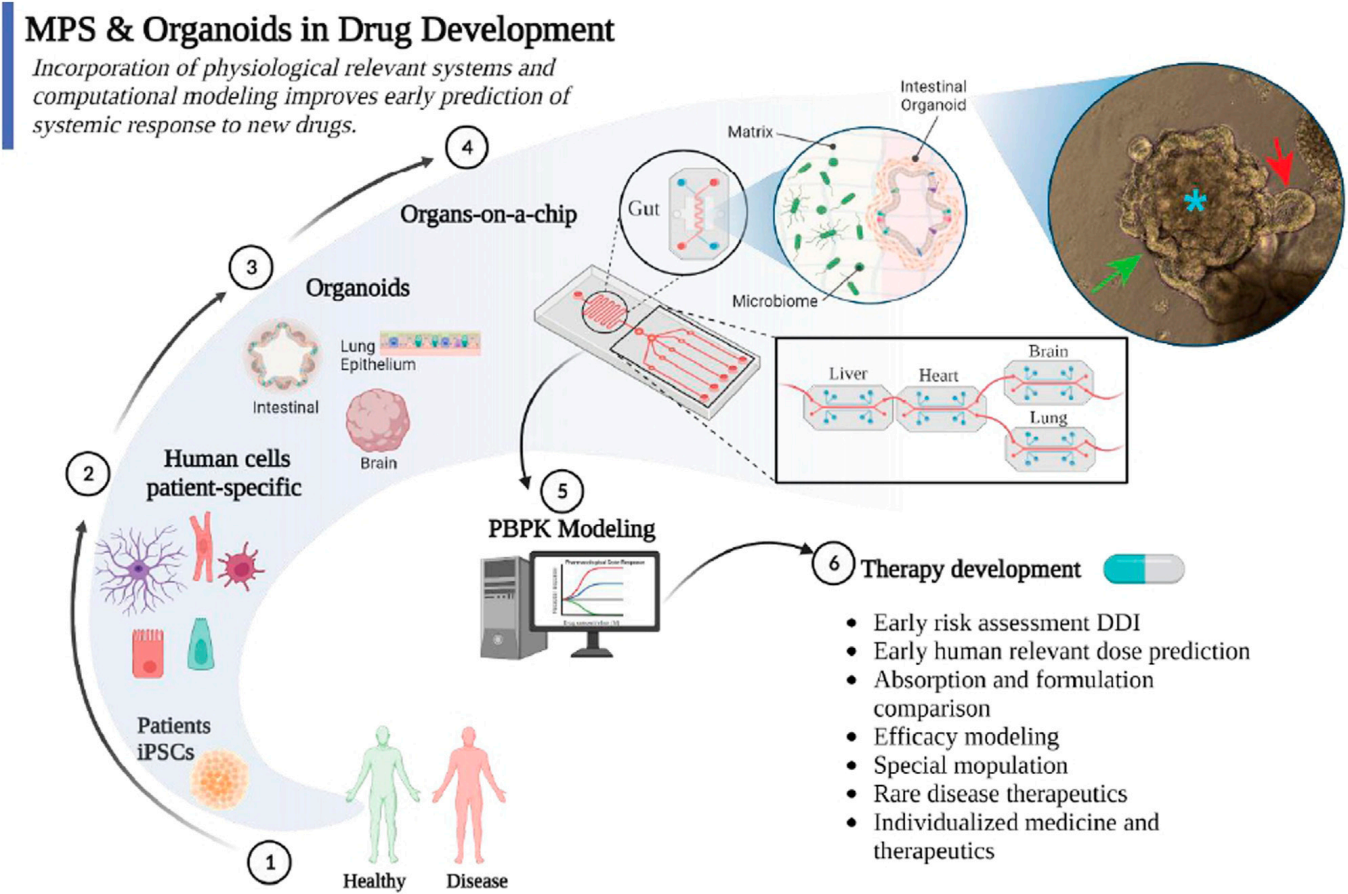
Integration of patient-specific organoids, such as intestinal organoids, into microphysiological systems (MPS) provides a powerful platform for early testing of new therapeutic compounds. Also, these *in vitro* platforms allow identification of pharmacokinetics parameters for the development of highly predictive pharmacokinetic models that improve the clinical translational of new drugs. The image depicts an example of iPSC derived intestinal organoids generated in Cristoforetti’s lab at University of Florida. Crypt-like domains (red arrow), lumen (blue star), and villus domain (green arrow).

Generating human organoids from iPSCs mimics the stages of the organ developmental process. Hence, for organs with long maturation timeframe such as brain, protocols to generate of iPSCS derived organoids might take over 60 days, while for fast regenerating organs like the intestines it can take about 7–10 days. Figure 1 shows an example of iPSC derived intestinal organoid cultured for 6 days. These organoids recapitulate the cellular hierarchy in the intestines with crypts-like domains, villus regions, and central lumen. Recently different systems, including breast cancer (Liu et al., 2022), pancreatic (Seppala et al., 2022), and gastrointestinal organoids (Rodrigues et al., 2022) have been successfully used for drug screening, response prediction, and toxicity studies. Furthermore, comparing the results using organoids with clinical outcomes demonstrated the advantage that organoids offer to speed up the generation of data to guide treatment and dose selection (Seppala et al., 2022). Altogether, these organoids provide a promising model for drug efficacy and toxicity screening. MPS are *in vitro* platforms containing human or animal cells/tissues within a biomimetic microenvironment to yield physiologically relevant mechanical, biochemical, and electrical responses (Rothbauer et al., 2021). As discussed by Feuilloley’s research group from the University of Rouen Normandie (Zommiti et al., 2022), MPS have significantly advanced from the original academic-only environment. The business model of many rising companies revolves around the commercialization of MPS (Ribas et al., 2018). These MPS range from *in vitro* reductionist models of a specific functional unit of an organ (e.g., 3D model of lung epithelium (Huang et al., 2013) to more complex single and multiple interconnected organs-on-chips (8 connected -organs-on-chips (Novak et al., 2020). MPS have demonstrated significant value for the drug development industry. For instance, systems like human 3D models of healthy (Hoffmann et al., 2018) and cystic fibrosis respiratory track (Nickolaus et al., 2020) have been used to identify optimal drug and dose to treat patients. While the use of MPS is questioned because it is highly complex structure, it often offers very high sensitivity and specificity. For instance, recent paper reported human-liver-chip with 100% specificity and 87% sensitivity when predicting drug-induced liver injury (Ewart et al., 2022). Despite different views about MPS and organoids as competing systems, integrating both as complementary technology is gaining popularity. Hence, the combination of MPS and organoids is in its infancy and carries an exciting future to accelerate pharmaceutical drug development by improving the clinical translation of basic research (Figure 1).

## Recent advances and future perspectives in the field of drug metabolism and transport

In this Research Topic we looked for articles exploring metabonomics to aid in drug development, as well as innovative tool design for analysis of drug transport and metabolism *in vitro* and *in vivo*. In this context, Ruan et al. used an acne vulgaris rat model to investigate the mechanism by which licorice flavonoids regulate skin metabolism, serum metabolism and skin microbes. Their results showed that licorice flavonoids could treat acne by regulating the metabolic balance of amino acids, lipids and fatty acids in serum and skin, which kept the microecology close to the normal skin state of rats. Peng et al. applied *in vitro* cell based and animal studies to investigate the impact of vitamin D deficiency on the exposure and response to pravastatin. The authors observed that vitamin D deficiency decreases the response of pravastatin in rats by reducing the liver pravastatin exposure and expression of hepatic OATPs, consistent with the extended hepatic clearance model theory. These results suggest that the impact of an OATP-based drug-drug interaction may differ depending on whether the individuals show vitamin D deficiency or not. Finally, Tao et al. used aspirin eugenol esther to illustrate the importance of concomitantly assessing drug metabolism and transport of prodrugs using *in vitro* cell-based methods. By improving the physiological relevance of the *in vitro* model, e.g., mimicking drug fate in the intestinal lumen, the authors were able to improve the predictability of the *in vitro* model. Altogether, the results of the articles presented in this Research Topic demonstrate that incorporating metabolomic analysis in physiological based models improves the prediction of drug response and drug-drug interactions.
